# Factors Affecting D-Lactic Acid Production by Flocculant *Saccharomyces cerevisiae* Under Non-Neutralizing Conditions

**DOI:** 10.3390/microorganisms13030618

**Published:** 2025-03-07

**Authors:** Dianti Rahmasari, Prihardi Kahar, Arthur Vinícius de Oliveira, Filemon Jalu Nusantara Putra, Akihiko Kondo, Chiaki Ogino

**Affiliations:** 1 Department of Chemical Science and Engineering, Graduate School of Engineering, Kobe University, Kobe 658-8501, Hyogo, Japan; dianti.rahmasari@gmail.com (D.R.); arthurolv20@gmail.com (A.V.d.O.); jalu@bear.kobe-u.ac.jp (F.J.N.P.); 2 Engineering Biology Research Center, Kobe University, 1-1 Rokkodai-cho, Nada-Ku, Kobe 657-8501, Hyogo, Japan; 3 Graduate School of Science, Technology, and Innovation (STIN), Kobe University, Kobe 658-8501, Hyogo, Japan; akondo@kobe-u.ac.jp; 4 Research Center for Membrane and Film Technology, Kobe University, Kobe 657-8501, Hyogo, Japan

**Keywords:** locus integration, flocculation, gene transcription, D-lactic acid, *Saccharomyces cerevisiae*

## Abstract

Integrating heterogeneous genes is widely used in metabolic engineering to produce D-lactic acid (D-LA), an essential compound in bioplastics and pharmaceuticals. However, research on the effects of integrating various loci on gene expression, especially regarding flocculation behavior, remains limited. This study constructed *Saccharomyces cerevisiae* strains by incorporating a codon-optimized *D-LDH* gene from *Leuconostoc pseudomesenteroides* (LpDLDH) into the specific genomic loci of the *CYB2*, *PDC1*, *MPC1*, *PDC6*, *ADH1*, and *PDC5* genes to redirect pyruvate toward lactic acid. Strains with the *LpDLDH* gene integrated at the *PDC1* locus achieved the highest D-LA titers (51 g/L) with minimal ethanol byproduct, followed by strains with integrations into the *CYB2* locus at 31.92 g/L, the *MPC1* locus at 10 g/L, and the *PDC6* locus at 0.026 g/L. In contrast, strains with *LpDLDH* integrated at the *ADH1* and *PDC5* loci failed to produce detectable levels of D-LA and exhibited a complete loss of flocculation. Gene expression analysis revealed a significant expression of genes related to flocculation (*FLO5*), stress adaptation (*HSP150*), and cell wall integrity (*YGP1*, *SED1*, and *SCW11*). The *CYB2*-integrating strain showed strong flocculant properties, contributing to its robustness. These findings highlight the influence of genomic locus selection on metabolic flux and stress adaptation, offering insights into optimizing D-LA production in flocculant *S. cerevisiae* yeast.

## 1. Introduction

The demand for lactic acid has increased significantly owing to its extensive application, particularly as a monomer for producing biodegradable and biocompatible polylactic acid materials. Consequently, enhancing fermentation processes to produce optically pure lactic acid has garnered substantial interest in recent research [1]. Lactic acid (LA) also serves as a key precursor in the production of various chemicals, including acrylic acid, acetaldehyde, pyruvic acid, 2,3-pentanedione, 1,2-propanediol, LA esters, and polylactic acid. Polymer-grade LA is essential for biomedical research and applications and serves as a fundamental raw material for the manufacture of environmentally friendly polylactic acid plastics. The diverse applications of LA underscore its importance as a highly valued product on the global market [2,3]. Blending poly D-LA with poly l-lactic acid through stereo-complexation results in plastics with enhanced thermal stability [4,5]. Large-scale L-lactic acid production by major chemical companies utilizing microbes has been well established [6]. However, limited research on producing D-lactic acid using microbes necessitates a more efficient method.

D-lactic acids are efficiently synthesized in substantial quantities by lactic acid bacteria, including *Lactobacillus*, *Sporolactobacillus*, and *Leuconostoc* [7]. For instance, *Sporolactobacillus inulinus* YBS1-5 produced 107.2 g/L of D-LA through fed-batch fermentation utilizing corncob residue hydrolysate, yielding 0.85 g/g of glucose equivalent [8]. Additionally, *Leuconostoc mesenteroides* B512 produced 60.2 g/L of D-LA through shake-flask fermentation using sugarcane juice, yielding 0.51 of glucose [9]. Typically, fermentation is effective at approximately neutral pH, is regulated using neutralizing agents, and produces lactate salts. This presents a challenge in the production process. However, the desired product for the polymerization reaction is undissociated (free) lactic acid, not its salt, necessitating further processing to recover the free lactic acid [4]. To address this issue and streamline production, fermentation without neutralizing agents and with a high yield of D-LA is required to simplify the process. However, this approach comes with its own set of challenges. While these bacteria are efficient producers of D-lactic acid, the growth of lactic acid bacteria is inhibited by a low pH due to high concentrations of lactic acid, resulting in the loss of viability and cellular activity [10], limiting their use in non-neutralizing fermentation conditions.

*Saccharomyces cerevisiae* exhibits a strong tolerance for acid and is suitable for the industrial-scale production of D-LA under non-neutralizing conditions [11]. Although *S. cerevisiae* does not naturally produce D-LA, optically pure D-LA can be synthesized through the heterologous expression of stereospecific D-lactate dehydrogenase (*D-LDH*). To achieve high-yield D-LA production in *S. cerevisiae*, the ethanol fermentation pathway is typically inactivated by disrupting the genes related to the activities of pyruvate decarboxylase (*PDC1*, *PDC5*, and *PDC6*) and alcohol dehydrogenase (*ADH1*), which control the initial steps of ethanol synthesis. Furthermore, to improve D-LA titers, it is essential to minimize D-LA consumption by disrupting the gene for mitochondrial inner membrane lactate dehydrogenase (*DLD1*), which oxidizes D-LA to pyruvate, along with the gene for mitochondrial intermembrane space cytochrome b2 (*CYB2*), which is vital for lactate utilization [11].

In addition to optimizing *D-LDH* integration loci, it is crucial to elucidate the physiological and molecular changes associated with D-LA production. No study has examined the relationship between D-LA production and flocculation traits in engineered strains with different locus integrations. The flocculation trait, a key characteristic of *S. cerevisiae* in industrial applications, is influenced by the intrinsic complexity of yeast flocculation regulation, which is governed by yeast genetics and environmental conditions [12]. The production of D-LA involves significant metabolic redirection and acid stress, which can affect cell wall integrity, stress response mechanisms, and flocculation phenotypes [13]. Investigating these changes through gene expression analysis can reveal the interplay between cell wall components, stress resistance pathways, and fermentation performance.

This research aims to expand existing knowledge by evaluating the flocculant *S. cerevisiae* yeast suitable for D-LA production and investigating the effects of genome integration loci on the *D-LDH* transgene expression cassette and D-LA production in this flocculant strain. It emphasizes how these loci influence traits related to flocculation and gene expression associated with cell wall integrity and stress response. Identifying optimal integration sites and elucidating the associated gene expression changes will facilitate more efficient microbial production of D-LA by the flocculant *S. cerevisiae* yeast strain and contribute to developing sustainable methods for producing biodegradable materials.

## 2. Materials and Methods

### 2.1. Strains and Medium

In this study, *S. cerevisiae* F118 (NBRC268), a flocculant strain obtained from the National Biological Resource Center (NBRC) culture collection in Tokyo, Japan, served as the primary host yeast for recombinant construction and fermentation. The *Escherichia coli* NEB stable (high efficiency) strain (New England Biolabs, NEB, Ipswich, MA, USA) was utilized for routine cloning. Specifically, *E. coli* was cultivated in Luria–Bertani (LB) medium, which contained 10 g/L of tryptone, 5 g/L of yeast extract, and 10 g/L of sodium chloride, supplemented with 100 µg/mL of ampicillin at 37 °C while shaking at 150 rpm. The yeast was grown in yeast extract peptone dextrose medium (YPD), consisting of 20 g/L of peptone, 10 g/L of yeast extract, and 100 g/L of glucose. Solid media for both *E. coli* and *S. cerevisiae* were prepared by adding 15 g/L of agar to the liquid medium. An appropriate antibiotic was supplemented into the medium depending on the selection markers used. Plasmid pAUR101 (TakaraBio Inc., Kusatsu, Japan) was employed to amplify transgene expression cassettes in *E. coli*, with the antibiotic ampicillin serving as a selection marker. For yeast fermentation, the antibiotics ampicillin and kanamycin were used to prevent *E. coli* contamination, while the antibiotic geneticin/G418 (Cat. No. ant-gn-1, InvivoGen, San Diego, CA, USA) was used to maintain positive recombinant offspring during successive subcultures. For comparative analysis, *S. cerevisiae* I231 (NBRC1226), J81 (NBRC1953), and J147 (NBRC103959) from the NBRC culture collection, and CCUG53310 sourced from the Culture Collection University of Gothenburg (Sweden) were also included as flocculant yeast strains for evaluation.

### 2.2. Construction of Transgene Expression System for D-Lactate Synthesis

**Figure 1:**
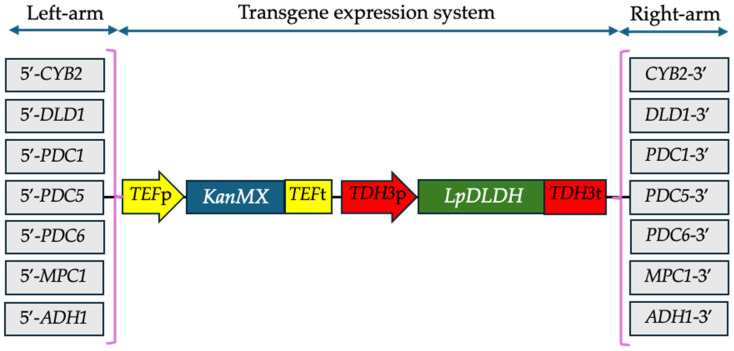
A transgene expression system based on the homologous recombination of the gene locus on the *Saccharomyces cerevisiae* chromosomes was used in this study.

To enable *S. cerevisiae* to produce D-LA from glucose, the expression system for the transgene cassettes involved in D-LA synthesis was constructed as follows (Figure 1). Initially, a codon-optimized *D-LDH* transgene from *Leuconostoc pseumesenteroides* (GenBank accession number: MW574953) was arranged in the expression cassette driven by the *TDH3* promoter and terminator. This transgene cassette was combined with the *KanMX* expression cassette to provide an antibiotic selection for the designed recombinant strains. Consequently, the positive recombinant strain could be selected and maintained for further investigation. To introduce the transgene expression cassettes into the chromosomes of the wild-type strain using homologous recombination techniques, left-arm and right-arm sequences containing 5′-*CYB2*, 5′-*DLD1*, 5′-*PDC1*, 5′-*PDC5*, 5′-*PDC6*, 5′-*MPC1*, and 5′-*ADH1* partial left sequences and 3′-*CYB2*, 3′-*DLD1*, 3′-*PDC1*, 3′-*PDC5*, 3′-*PDC6*, 3′-*MPC1*, and 3′-*ADH1* partial right sequences from the *S. cerevisiae* chromosomes were cloned from the F118 strain genome by PCR method using the primer sets listed in Supplementary Table S1. These were infused with transgene expression cassettes, as shown in Figure 1, resulting in their genome integration into the *CYB2*, *DLD1*, *PDC1*, *PDC5*, *PDC6*, *MPC1*, and *ADH1* loci. The transgene expression cassettes bearing only the KanMX expression cassette were designed using the primer sets provided in Supplementary Table S2 for constructing disrupted strains lacking *D-LDH*, which served as negative controls in this study. The linear nucleotides were subsequently transformed into yeast cells following the lithium acetate/single-stranded carrier DNA/polyethylene glycol method [14]. Transformants were obtained by incubating the cells on YPD agar with 200 mg/L of geneticin at 30 °C for 3 to 5 days. The transgene expression cassette, which includes the left and right arms of the locus, was inserted into the SmaI restriction site of pAUR101 and transformed into *E. coli* for further amplification and purification prior to the yeast transformation.

### 2.3. Fermentation

A single colony of the yeast strain grown on YPD agar was selected and inoculated into 12 mL of YPD100 medium (100 g/L of glucose) in a 100 mL Erlenmeyer flask. The cultures were incubated at 150 rpm for 1 to 2 days to serve as a seed culture. To initiate the main culture, the seed was inoculated into 12 mL of YPD with 100 g/L of glucose (YPD100) and cultivated at 90 rpm until the glucose in the medium was depleted. After harvesting the cells from the seed culture, the initial cell concentration was adjusted to OD_600nm_ = 5 to ensure that the F118 WT strain, the primary host yeast, could be conditioned for early exponential growth, initiating fermentation and completely consuming 100 g/L of glucose within 24 h. The experiments were conducted with three biological replicates, and the cultivation time varied depending on the strain, ranging from 24 to 144 h.

### 2.4. Fermentation Product Measurements

Several compounds related to the fermentation, including glucose, D-LA, glycerol, acetic acid, and ethanol in the fermentation samples, were analyzed using high-performance liquid chromatography (HPLC) equipped with a refractive index detector (RID-10A, Shimadzu, Kyoto, Japan). A Coregel-87H column (7.8 mm ID × 300 mm, Transgenomic Inc., New Haven, CT, USA) was used at 80 °C with 5 mM sulfuric acid as the eluent at a 0.6 mL/minute flow rate for 40 min. Fermentation samples were periodically collected and then centrifuged at 14,000× *g* for 5 min at 4 °C. The supernatants were collected and filtered through a 0.45 μm polytetrafluoroethylene (PTFE) filter (Merck Millipore, Carrigtwohill, Ireland) into HPLC vials.

### 2.5. Cell Growth Measurement

The growth of the yeast was assessed by measuring the dry cell weight, as the flocculent F118 strains could not be evaluated using optical density. At the start (0 h) and end of batch fermentation, cultures from each sample were centrifuged at 14,000× *g* for 5 min at 4 °C to harvest the cell pellets, which were washed twice with sterilized water. The pellets were then stored at −80 °C and freeze-dried overnight. Finally, the dry cell weight was measured.

### 2.6. Cell Morphological Observation

The morphological changes in the cells resulting from gene disruption and the expression of *LpDLDH* transgene cassettes in the F118 strain were observed using a BIOREVO BZ-9000 digital microscope (Keyence, Osaka, Japan) at the magnification power of a ×100 objective lens.

### 2.7. Cell Wall Hydrophobicity Test

The cell wall hydrophobicity of the cultivated cells was determined using a modified microbial adhesion to hydrocarbon assay [13,15]. The cells were deflocculated with 50 mM ethylenediaminetetraacetic acid (EDTA, pH 7) and resuspended in 2 mL of 0.9% sodium chloride solution. The initial absorbance (*A*_0_) was measured at 600 nm using a spectrophotometer (UVmini-1240, Shimadzu). Subsequently, 0.4 mL of octane was added to the cell suspension, and the mixture was vortexed at maximum speed for 60 s. After standing at room temperature for 10 min, the aqueous phase containing non-hydrophobic cells was separated, and its absorbance (*A*_1_) was measured at 600 nm. The cell wall hydrophobicity was calculated using Formula (1).

(1) $$\mathrm{Cell}\;\mathrm{wall}\;\mathrm{hydrophobicity}=(1-\frac{\;A_{1}}{A_{0}}\times 100)$$

### 2.8. Quantitative PCR for Gene Expression Analysis

Yeast cells were collected during the mid-exponential phase of aerobic fermentation, as this is when cells show their peak metabolic activity. Conducting gene expression analysis at this stage reduces the impact of stationary-phase stress responses and offers insights into how metabolic pathways are regulated under optimal growth conditions. In some cases, gene expression changes were assessed after 6 h of cultivation to capture early metabolic shifts. At this point, cells actively metabolize glucose, and the expression of genes involved in D-LA biosynthesis can be observed before significant environmental stress or nutrient depletion affects transcriptional regulation. To do that, a volume of 0.5–1 mL of cell suspension was centrifuged at 8000 × *g* for 10 min at 4 °C. The pellets were washed once with cold sterilized water and stored at -80 °C until RNA extraction. RNA was extracted using the NucleoSpin RNA kit (Macherey-Nagel, Düren, Germany), with DNA contamination minimized by rDNase treatment during column digestion, following the manufacturer’s instructions. Reverse transcription was performed using ReverTra Ace qPCR RT Master Mix with gDNA remover (Toyobo, Osaka, Japan), and the resulting cDNA was used for quantitative PCR (qPCR). Gene expression levels were quantified using KOD SYBR qPCR Mix (Toyobo) with 0.4 µM of each forward and reverse primer and 10 ng/µL of diluted cDNA. qPCR was conducted on a Mx3005P system (Agilent Technologies Ltd., Tokyo, Japan). The PCR program included an initial denaturation at 95 °C for 10 min, followed by 60 cycles of 30 s at 95 °C, one minute at 55 °C, and one minute at 72 °C for elongation. The final step included one cycle of one minute at 95 °C, 30 s at 55 °C, and 30 s at 95 °C.

The *ACT1* gene was used as an internal reference due to its stable expression across all samples, with consistent Ct values. In some cases, the *TUB1* gene was used as an internal reference instead of the *ACT1* gene. The expression levels of target genes were calculated as fold changes using the formula 2^−ΔΔCt^, where ΔΔC_t_ = ΔC_t_ sample − ΔC_t_ control and ΔC_t_ sample = C_t-sample_ − C_t-ACT1_ and based on calculations from the MxPro QPCR software v4.10 (Agilent Technologies Ltd., Tokyo, Japan). Primer sequences used in the analysis were designed using the NCBI Primer-BLAST tool (http://www.ncbi.nlm.nih.gov/tools/primer-blast/) (accessed on 1 March 2025), with a target product size of approximately 100 bp, as listed in Supplementary Table S3.

### 2.9. Statistical Analysis

All experiments on cultivations were conducted in a single independent cultivation with three technical replicates per strain. The mean and standard deviation (SD) were calculated for each dataset. Statistical significance between strain groups of D-LA-producing strains and disrupted strains was determined using an unpaired two-tailed Student’s *t*-test. A *p*-value of <0.05 was considered statistically significant.

### 2.10. Differential Expression Analysis

Differential gene expression analysis was conducted using the DESeq2 package in *R* (version 4.4.2). Count data were imported into DESeq2, and genes with fewer than 10 total counts across all samples were filtered out to reduce noise. The count matrix was normalized using DESeq2’s median of ratios method to account for differences in sequencing depths across samples.

Statistical testing was performed using the DESeq function, with a negative binomial model used to estimate fold changes and significance. Genes were considered significantly differentially expressed (DEGs) if they met the following criteria: adjusted *p*-value (*P*_adj_) < 0.05 and absolute log_2_ fold change (Log_2_FC) > 1. DEGs were visualized using a volcano plot, highlighting significantly upregulated and downregulated genes. The volcano plot was generated in R to visualize the distribution of DEGs, with upregulated genes (Log_2_FC > 1, *P*_adj_ < 0.05) highlighted in red and downregulated genes (Log_2_FC < −1, *P*_adj_ < 0.05) highlighted in blue. Genes that did not meet the significance thresholds are shown in black.

## 3. Results

### 3.1. D-LA Production Profiles by Engineered Flocculant S. cerevisiae Strains

To evaluate D-LA production by engineered flocculant *S. cerevisiae* strains, the strains were cultivated in Erlenmeyer flasks with the medium containing 100 g/L of glucose at 150 rpm and 30 °C until glucose depleted. Since the strains have a strong flocculation trait, the initial cell concentration could not be adjusted accurately; therefore, the same wet cell weight (approximately equal to OD_600nm_ = 5) was used, as mentioned in Section 2.3.

**Figure 2:**
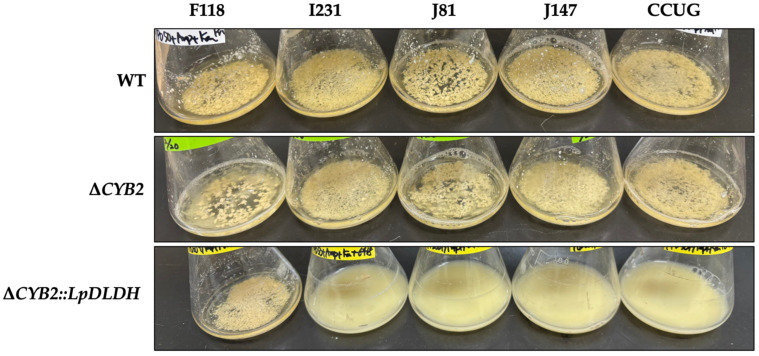
Changes in phenotype resulting from integrating the *LpDLDH* gene expression system involved in lactic acid synthesis into flocculating *S. cerevisiae* strains.

As shown in Figure 2, all WT strains exhibit a flocculation trait, with the F118 strain demonstrating more pronounced flocculation behavior than the others. The disruption of the *CYB2* gene in all strains (Δ*CYB2*) does not influence the flocculation traits of the strain. However, after implementing the transgene expression system for D-LA production at the *CYB2* locus in their chromosomes, the recombinant F118 strain (Δ*CYB2*::LpDLDH) retained the flocculation trait, albeit to a lesser degree compared to the WT strain.

In contrast, the other recombinant strains (I231Δ*CYB2*::*LpDLDH*, J81Δ*CYB2*::*LpDLDH*, J147Δ*CYB2*::*LpDLDH*, and CCUGΔ*CYB2*::*LpDLDH*) lost their flocculation traits. The loss of flocculation traits indicates a cellular response to metabolic stress caused by integrating the D-LA synthesis pathway. Typically, disrupting the *CYB2* gene does not impact *S. cerevisiae* cell growth or phenotype, as its expression is generally suppressed during glucose fermentation. Similarly, *CYB2* gene expression decreases under anaerobic conditions while producing lactic acid [16,17]. Research by Pangestu et al. [18] demonstrated that the recombinant F118 strain (Δ*CYB2*::*LcLLDH*), which utilized the L-LA production system employing *L-LDH* from *Lactobacillus casei*, preserved its flocculation behavior without any decline. This finding was corroborated by the upregulation of flocculation-related genes such as *FLO5* and genes associated with cell wall hydrophobicity, including *YGP1* and its *Haa1* regulon.

**Figure 3:**
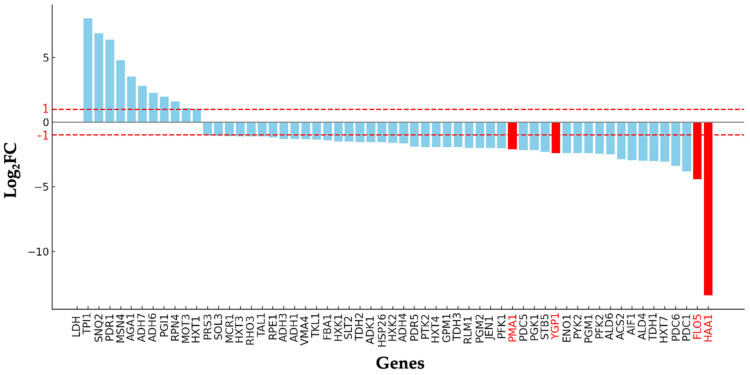
Changes in expression of significant genes involved in lactic acid fermentation, comparing the Δ*CYB2* and the Δ*CYB2*::*LcLLDH* strains after 6 h of cultivation in the YPD100 medium without a neutralizer addition.

**Figure 4:**
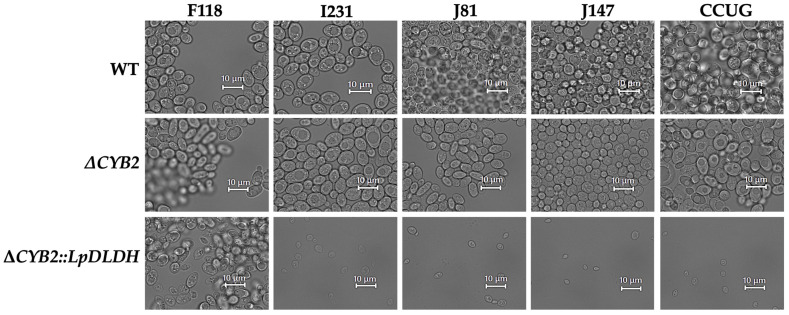
Morphological changes in the shape of the WT, the Δ*CYB2*, and the Δ*CYB2*::*LpDLDH* strains after 48 h of cultivation in the YPD100 medium without neutralizer. See more in Supplementary Figure S1.

In contrast, these genes (*FLO5* and *HAA1*) are downregulated during the production of D-LA, as illustrated in Figure 3. The *PMA1* gene, also downregulated by the *Haa1* regulon, encodes the H(+)-ATPase *Pma1* on the yeast plasma membrane, which expels protons dissociating from weak acid molecules [19]. *Pma1* is crucial for yeast adaptation to weak acids, generating an electrochemical proton gradient vital for nutrient uptake and the regulation of intracellular pH [20,21]. Furthermore, the *Haa1* regulon significantly contributes to the rapid adaptation of yeast to weak acids, regulating approximately 80% of weak acid-induced gene expressions, both directly and indirectly [22,23]. This indicates that the substantial decrease in *HAA1* gene expression observed (Figure 3) contributed to the compromised condition of F118 cells and reduced flocculation levels. Recently, the flocculation behavior of the F118 strain was altered in response to the concentration of inhibitory chemical compounds (ICCs) in the culture medium. This study indicated that Mot3p was involved in this response mechanism based on its range of prionogenicity [13]. In this study, the expression of the *MOT3* gene was nearly identical to that of the Δ*CYB2* strain. However, the expression levels of the *FLO5* and *YGP1* genes, which are associated with flocculation and modified cell wall hydrophobicity, respectively, decreased. This suggests that the D-LA produced within the F118 cells triggered a different ICC response than those ICCs present outside the cells. This contrasts with the previously reported phenomenon of L-LA production in the Δ*CYB2* strain [18]. This indicates that D-LA is more toxic than L-LA for yeast cells despite the fact that the F118 strain is more resilient than the other evaluated strains. The loss of flocculation traits in recombinant strains (I231Δ*CYB2*::*LpDLDH*, J81Δ*CYB2*::*LpDLDH*, J147Δ*CYB2*::*LpDLDH*, and CCUGΔ*CYB2*::*LpDLDH*) might be due to low expressions of the *FLO5* and *YGP1* as well as *MOT3* genes in these WT strains. Consequently, the detrimental effect on the flocculation traits and their resilience during the fermentation of D-LA was more pronounced than in the F118 strain, as shown in Figure 4. The disruption of *CYB2* in all strains (Δ*CYB2*) has no significant effect on shape and aggregation properties, as the WT and Δ*CYB2* strains are comparable. However, integrating the *LpDLDH* transgene expression cassette into the *CYB2* locus significantly impacts the morphological and aggregation properties of the I231, J81, J147, and CCUG strains. The strain cells decrease in size compared to the WT strains and lose their flocculation traits, except for the F118 strain.

**Figure 5:**
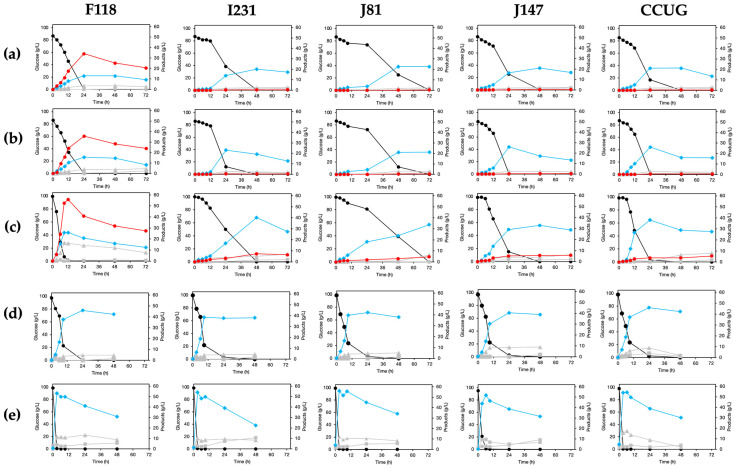
D-lactic acid production by F118Δ*CYB2*::*LpDLDH*, I231Δ*CYB2*::*LpDLDH*, J81Δ*CYB2*::*LpDLDH*, J147Δ*CYB2*::*LpDLDH*, and CCUGΔ*CYB2*::*LpDLDH* (**a**–**c**) compared to that by the Δ*CYB2* (**d**) and WT strains (**e**), with a YPD medium containing 100 mM MES/NaOH buffer (pH of 5.5) and glucose under the presence of CaCO_3_ as a neutralizer (**a**), without a neutralizer (**b**), and YPD without 100 mM MES/NaOH buffer and a neutralizer (**c**). Black closed circle: glucose consumption; blue closed diamond: ethanol; red closed circle: D-LA; gray closed triangle: glycerol; gray closed box: acetic acid.

During fermentation, the detrimental effects of weak acids, such as lactic acid, lead to reduced glucose consumption and lower fermentation rates for D-LA production (Figure 5). To verify these effects, fermentations were conducted in a YPD medium containing glucose as a carbon source buffered with MES/NaOH (pH of 5.5, similar to the initial pH) at 100 mM, with or without CaCO_3_ at 5 g/L, which served as a neutralizer for the lactic acid released into the medium. Consequently, glucose consumption occurred more rapidly when the F118, J147, and CCUG strains were cultivated in a medium without buffering to a pH of 5.5 and without CaCO_3_ neutralization (a < b < c), indicating that these strains are more resilient than the I231 and J81 strains during D-LA production under these conditions. A similar trend was observed in D-LA production by the recombinant strain of F118 (Δ*CYB2*::*LpDLDH*), which achieved 56 g/L of D-LA after 12 h of fermentation, yielding 0.56 g of D-LA/g-glucose consumed. Interestingly, the recombinant strains of I231Δ*CYB2*::*LpDLDH*, J81Δ*CYB2*::*LpDLDH*, J147Δ*CYB2*::*LpDLDH*, and CCUGΔ*CYB2*::*LpDLDH* failed to produce D-LA when cultivated in the medium buffered to a pH of 5.5 and neutralized with CaCO_3_. These strains were ultimately capable of producing D-LA when grown in the medium without buffering and CaCO_3_ neutralization, though the yield was very low (less than 0.05 g of D-LA/g-glucose consumed). In contrast, ethanol was primarily produced, differing from the culture of strain F118. Thus, the detrimental effects of weak acids, such as lactic acid, on glucose consumption and D-LA production have been clarified, revealing a significant causal relationship with changes in flocculation traits.

In this study, the robust constitutive *TDH3* promoter was utilized to enhance the high expression of the *LpDLDH* gene in all engineered strains under elevated glucose conditions. However, its activity diminishes in acidic environments. Once D-LA is produced and released into the medium, extracellular lactic acid can diffuse through the plasma membrane into the cytosol, dissociating into lactate and protons, which promotes intracellular acidification. This results in a decrease in D-LA yield due to reduced cell growth and the repression of *TDH3* promoter activity [24]. Nevertheless, the F118Δ*CYB2*::*LpDLDH* strain can achieve a high yield of D-LA because it maintains the flocculation trait during fermentation. In contrast, the other flocculating strains (I231Δ*CYB2*::*LpDLDH*, J81Δ*CYB2*::*LpDLDH*, J147Δ*CYB2*::*LpDLDH*, and CCUGΔ*CYB2*::*LpDLDH*) could not sustain this trait even when the D-LA concentration was low. Consequently, D-LA may diffuse into the cytosol and lower the intracellular pH. This observation indicates that the flocculation trait is crucial for the yeast strains’ ability to tolerate weak acids and support effective lactic acid production, even in conditions lacking a buffering effect and without neutralization. The superior performance of strain F118 under challenging conditions highlights its potential as a strong platform for industrial-scale D-LA production.

### 3.2. Ideal Integration Spot of D-LDH in Flocculant S. cerevisiae F188 Strain for D-LA Production

**Figure 6:**
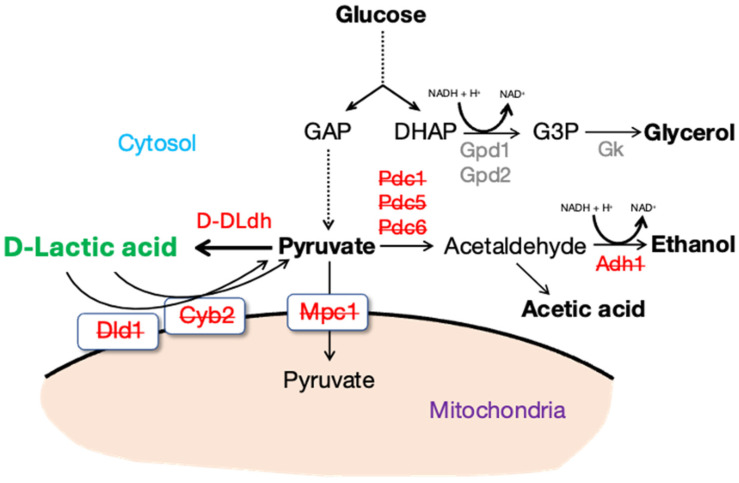
D-LA biosynthetic pathway in engineered *S. cerevisiae* highlights the key metabolic disruptions examined in this study. The red font with strikethrough indicates the targets for gene disruption, while the red font without strikethrough indicates the target for gene insertion.

The ideal genomic integration sites were considered to optimize *D-LDH* integration into the F188 strain for D-LA production. The engineered strains were developed to enhance D-LA production by altering the interactions between the ethanol and D-LA pathways (Figure 6). Research focused on lactate-oxidizing enzymes (*DLD1*, *CYB2*) has consistently shown higher D-LA yields in engineered yeast strains [25]. Pyruvate decarboxylases, encoded by *PDC1* and *PDC5*, convert pyruvate into acetaldehyde during ethanol fermentation. In *S. cerevisiae*, most of the catalytic activity for converting acetaldehyde into ethanol is attributed to the cytosolic alcohol dehydrogenase (*ADH1*) gene [26]. Disrupting these genes results in pyruvate accumulation, increasing the substrate pool for *D-LDH* to convert pyruvate into D-LA. Lactate production has risen dramatically in strains with *PDC* gene deletions that overexpress *D-LDH* due to the greater availability of pyruvate [27].

*Mpc1*, an enzymatic component of the mitochondrial pyruvate transport system, plays a significant role in this process. Inactivating the *MPC1* gene that encodes *Mpc1* results in the cytosolic retention of pyruvate, increasing its availability for *D-LDH*-mediated conversion to D-LA. Studies on mitochondrial pyruvate transport systems have revealed that disrupting the *MPC1* gene leads to elevated cytosolic pyruvate levels, promoting increased lactate production. The literature indicates that the absence of the *MPC1* gene is expected to cause the most significant reduction in mitochondrial pyruvate uptake, as the formation of both active carrier complexes (MP_COX_ and MPC_FERM_) is hindered without the *Mpc1* subunit [28].

In this study, the F118 strain was engineered to incorporate a transgene expression system for D-LA synthesis into the *CYB2*, *PDC1*, *DLD1*, *ADH1*, *PDC5*, *PDC6*, and *MPC1* loci, as outlined in Section 2.1 (Figure 1). The integration of the *D-LDH* transgene expression cassette into the various chromosomal loci of the F118 strain was achieved through homologous recombination. Subsequently, evolved strains were assessed for their ability to produce D-LA and their metabolic characteristics based on the locus of the *D-LDH* transgene expression cassette in the chromosomes.

**Figure 7:**
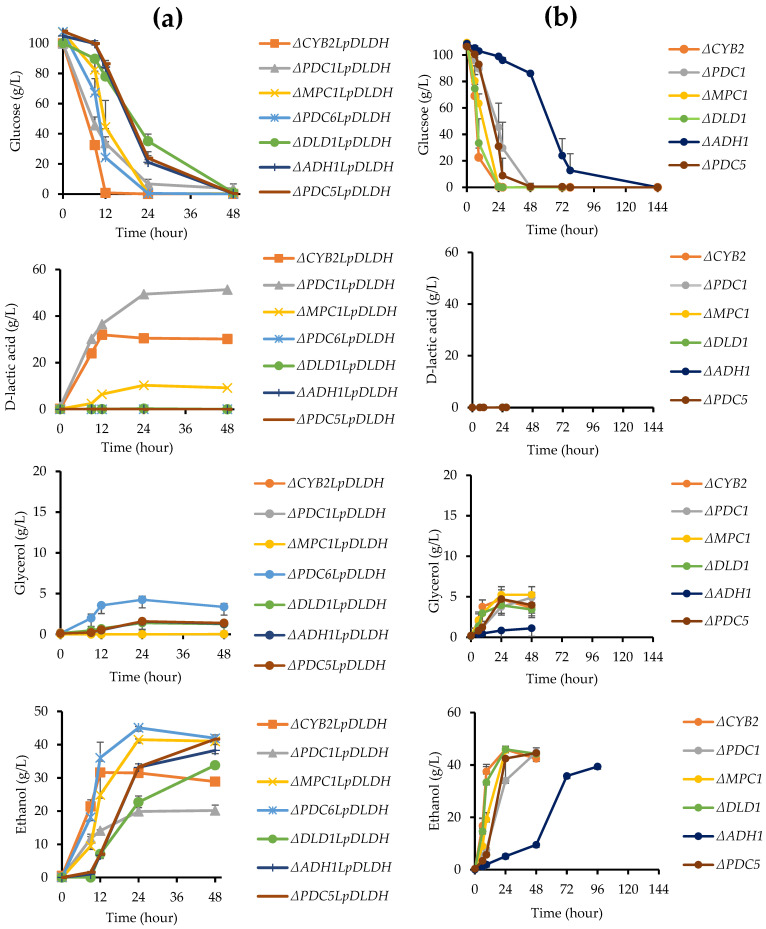
Fermentation profile of engineered F118 strains with *LpDLDH* (**a**) and without the integration of the *LpDLDH* transgene expression system (**b**), successfully grown in YPD media containing 100 µg/mL of geneticin as a selectable marker. The graph illustrates glucose consumption (g/L), D-lactic acid (g/L), glycerol, and ethanol production (g/L) across various strains with different locus integrations. Data points represent the mean of biological replicates, with error bars showing standard deviation.

D-LA production was performed using a high glucose concentration of 100 g/L to maximize the D-LA titer. The strain with the *D-LDH* transgene expression cassette integrated at the *PDC1* locus (Δ*PDC1*::*LpDLDH*) showed the highest D-LA titer, reaching 51.36 g/L, while the strain at the *CYB2* locus (Δ*CYB2*::*LpDLDH*) produced 31.92 g/L (Figure 7a). Moreover, the formation of ethanol byproducts was lower in the Δ*PDC1*::*LpDLDH* strain compared to the Δ*CYB2*::*LpDLDH* strain, with a reduction of approximately 35%, indicating a successful swap or disruption of the *PDC1* gene, which plays a central role in the ethanol pathway. These findings highlight the critical influence of the integration locus on the balance between D-LA production and ethanol byproduct formation, with the *PDC1* locus offering a more favorable environment for efficient D-LA synthesis in the F118 strain. Strains with *D-LDH* integrated at the *ADH1* locus (Δ*ADH1*::*LpDLDH*) or the *PDC5* locus (Δ*PDC5*::*LpDLDH*) successfully grew on the media containing G418 as a selection marker and consumed the glucose during fermentation by 48 h. However, they did not produce D-LA, indicating that the transgene expression system for the *D-LDH* gene, despite being integrated, may not have been functional or active enough to facilitate D-LA production.

Notably, the strain with *D-LDH* integrated at the *PDC6* locus (Δ*PDC6*::*LpDLDH*) or the *MPC1* locus (Δ*MPC1*::*LpDLDH*) was able to grow on the selective media. It produced D-LA, indicating that these integrations were sufficient for transformation and *D-LDH* gene expression. The Δ*PDC6*::*LpDLDH* and Δ*MPC1*::*LpDLDH* strains produced D-LA at lower titers of 10 g/L and 0.026 g/L, respectively. Both strains consumed glucose within 24 h and produced ethanol at 45 g/L and 41 g/L, respectively, comparable to the WT strain at 40 g/L. The high ethanol production in these strains suggests an incomplete redirection of the pyruvate flux toward D-LA synthesis, potentially due to the insufficient expression of *D-LDH* or the dominance of competing pathways.

The disrupted F118 strains lacking *D-LDH* (∆*CYB2*, ∆*PDC1*, ∆*DLD1*, ∆*ADH1*, ∆*PDC5*, ∆*PDC6*, and ∆*MPC1*), which served as negative controls (Figure 7b), exhibited varying growth rates, glucose consumption, and ethanol production. Notably, the ∆*CYB2*, ∆*DLD1*, and ∆*MPC1* strains displayed the fastest growth among the strains, fully consuming glucose within 24 h. These strains also demonstrated similar ethanol production levels, reaching 45.97 g/L, 46.04 g/L, and 45.72 g/L, respectively. In contrast, the ∆*PDC5* and ∆*PDC1* strains showed slower growth, with glucose consumption delayed until 48 h. The ∆*ADH1* strain experienced the slowest growth, taking approximately 144 h to completely consume glucose. Notably, the ∆*PDC6* strain could not utilize glucose, with levels remaining around 100 g/L even after 48 h without cell growth. These results highlight the significant differences in growth and metabolic efficiency among the disrupted strains, indicating variations in growth and metabolic efficiency. The ∆*CYB2*, ∆*DLD1*, and ∆*MPC1* strains demonstrated higher metabolic activities and ethanol production, while ∆*ADH1* and ∆*PDC6* exhibited impaired glucose utilization.

Based on these results, the *CYB2* loci can serve as targets for integrating the *D-LDH* transgene expression system in the F118 strain without compromising cell growth and metabolic activity. These findings underscore the potential for the further optimization of D-LA production in the F118 strain. Furthermore, the multi-integration of the *D-LDH* transgene expression system into *PDC1* and *CYB2* may enhance D-LA production. The engineered strains effectively produce D-LA even at high glucose concentrations. Since the expression of the *D-LDH* transgene in these strains is driven by the *TDH3* promoter, it is positively influenced by the glucose concentration in the medium. Catabolite repression occurs during D-LA production to prevent the utilization of D-LA and other byproducts, such as acetic acid and ethanol, as alternative carbon sources instead of glucose. Additional integration into the *ADH1* loci or the disruption of this gene to reduce ethanol byproduct formation is not recommended, as it may severely impact cell growth and metabolic activities, leading to low titers of D-LA production.

### 3.3. Flocculation Behavior Across the Engineered Strains

**Figure 8:**
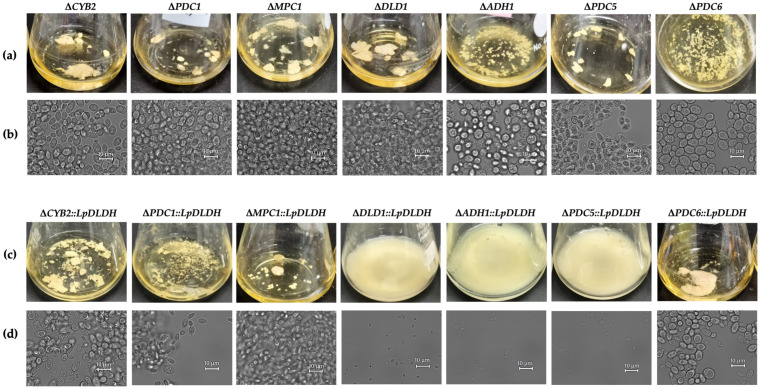
Flocculation behaviors of the F118 strain with locus destruction only (**a**,**b**) and concomitantly with the integration of the *LpDLDH* transgene expression cassette (**c**,**d**) after 48 h of cultivation in the YPD100 medium without neutralizer, and the morphological changes on the shape of cells are confirmed by using bright light mode (**b**,**d**). See more in Supplementary Figure S2.

To observe the effect of the robustness of the strain, flocculation was analyzed during the stationary phase compared to the WT strain, which functioned as a strong flocculant strain. The Δ*CYB2*::*LpDLDH* strain exhibited significant clumping, leading to the formation of large cell aggregates in the culture broth, while the Δ*PDC1*::*LpDLDH* strain showed less pronounced flocculation, resulting in smaller clumps (Figure 8c). These observations related to dry cell weight measurements for the Δ*PDC1*::*LpDLDH* strain, which indicated the lowest weight at 2.97 g/L. These differences highlight the variations in flocculation behavior between the two strains, with the Δ*CYB2*::*LpDLDH* demonstrating a higher degree of flocculation and larger cell aggregates. Meanwhile, the Δ*PDC1*::*LpDLDH* strain showed a lower, yet still evident, level of flocculation. Despite the discrepancies in flocculation behavior, the Δ*CYB2*::*LpDLDH* and the Δ*PDC1*::*LpDLDH* strains exhibited high hydrophobicity values (Table 1). Additionally, Δ*PDC6*::*LpDLDH* and Δ*MPC1*::*LpDLDH* presented large clumps of cells. Notably, the strain Δ*PDC6*::*LpDLDH* displayed the highest dry cell weight. This indicates that cell surface hydrophobicity does not primarily drive the differences in flocculation observed between the strains, as both exhibited high hydrophobicity regardless of their aggregation patterns. Considering the high hydrophobicity noted in both strains, the reduced flocculation in the Δ*PDC1*::*LpDLDH* strain may relate to genetic factors rather than surface hydrophobicity, as mentioned in Section 3.1.

In contrast, strains with *D-LDH* integrated into the *DLD1* (Δ*DLD1*::*LpDLDH*), *ADH1* (Δ*ADH1*::*LpDLDH*), and *PDC5* (Δ*PDC5*::*LpDLDH*) loci, which failed to produce D-LA, were observed to be completely non-flocculant. These strains showed no visible clumping, and the cells remained evenly dispersed throughout the culture, indicating a loss or absence of flocculation behavior. Interestingly, the disrupted strains lacking *D-LDH* (∆*CYB2*, ∆*PDC1*, ∆*DLD1*, ∆*ADH1*, ∆*PDC5*, ∆*PDC6*, and ∆*MPC1*), which served as negative controls (Figure 8a), still possessed the strong flocculation trait, similar to the WT strain; however, only the ∆*ADH1* and ∆*PDC6* strains exhibited the weak flocculation trait compared to the other strains.

The disruption of the locus genes in all strains (Δ*CYB2*, Δ*PDC1*, Δ*PDC6*, and Δ*MPC1*) has no significant effect on cell aggregation properties, as comparable to the WT strain, as shown in Figure 8b. However, integrating the *LpDLDH* transgene expression cassette into the *DLD1*, *ADH1*, and *PDC5* loci significantly impacts the morphological and aggregation properties of the Δ*DLD1*::*LpDLDH*, Δ*ADH1*::*LpDLDH*, and *ΔPDC5*::*LpDLDH* strains. The strain cells decrease in size compared to the Δ*DLD1*, Δ*ADH1*, and Δ*PDC5* strains and lose their flocculation traits, as shown in Figure 8d.

### 3.4. Gene Expression Analysis of D-LA-Producing Strains

A gene expression analysis was used to investigate mechanisms influencing lactic acid fermentation. *TUB1* and *ACT1* genes were evaluated across yeast strains using stability assessment tools to select an appropriate housekeeping gene for qPCR normalization. The chosen gene was used to normalize expression data for target genes related to D-LA synthesis and strain robustness. qPCR experiments were conducted and analyzed to identify differentially expressed genes. Gene expression patterns should be correlated with fermentation profiles and D-LA production levels to elucidate underlying mechanisms.

The results of the Ct values of housekeeping genes are listed in Supplementary Table S4. Results indicated that the *ACT1* gene exhibited more consistent Ct values, making it a more reliable reference gene. In contrast, the *TUB1* gene displayed variability, with a Ct value of 27.73 in the Δ*PDC1*::*LpDLDH* strain, three cycles higher than in the WT strain. The strain capable of producing D-LA showed low Ct values, which is beneficial as it confirms the presence of mRNA levels for the housekeeping genes. In contrast, the non-producing strain exhibited high Ct values, indicating potential issues. This difference suggests a problem with the non-producing strain, as *ACT1* or *TUB1*, being essential housekeeping genes, should usually be expressed in yeast cells; therefore, gene expression analysis can compare the D-LA producing strains (Δ*CYB2*::*LpDLDH*, Δ*PDC1*::*LpDLDH*, Δ*MPC1*::*LpDLDH*, and Δ*PDC6*::*LpDLDH*) to control (WT strain) to eliminate the backgrounds of related gene expressions after disruption.

**Figure 9:**
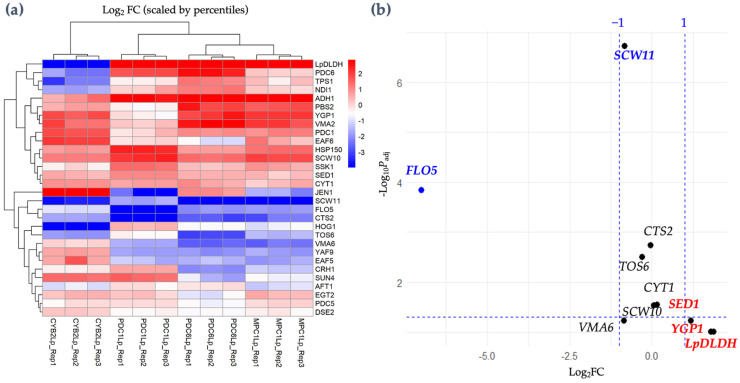
Relative expression levels of D-LA-producing strains (**a**) and volcano plot of differential expression across D-LA-producing strains (**b**). A volcano plot was generated to visualize the fold changes (log_2_FC) and statistical significance (*P*_adj_) of the genes. The analysis focused on genes with a *P*_adj_ < 0.05 and |log_2_FC| > 1 as thresholds for significant differential expression. Downregulated genes (represented in blue) have negative log_2_ fold changes (log_2_FC < 0). Non-significant genes are shown in black.

The heatmap analysis (Figure 9a) shows the expression levels of 30 genes across four strains with varying D-LA production levels, as described in the previous section (Figure 7, Table 1). Among these, the *LpDLDH* gene, encoding the *D-LDH*, exhibits significantly higher expressions in the Δ*PDC6*::*LpDLDH*, Δ*MPC1*::*LpDLDH*, and Δ*PDC1*::*LpDLDH* strains (red color). However, the Δ*CYB2*::*LpDLDH* strain displays a notably low Log_2_FC in *LpDLDH* expression despite producing 30 g/L of D-LA.

We also examined the gene expression changes associated with flocculation behavior by focusing on the *FLO5* gene. We evaluated the expression levels of *FLO5* across various engineered strains, including Δ*CYB2*::*LpDLDH*, Δ*PDC1*::*LpDLDH*, Δ*MPC1*::*LpDLDH*, and Δ*PDC6*::*LpDLDH*, which have previously been shown to produce different levels of D-LA. The heatmap data revealed significant differences in *FLO5* expression among the strains. Notably, the Δ*CYB2*::*LpDLDH* strain showed no substantial changes in *FLO5* expression compared to the other engineered strains, suggesting that the flocculation behavior in this strain was not significantly influenced by the integration of the D-LDH gene at the *CYB2* locus. In contrast, other strains exhibited the downregulation of *FLO5*, with the Δ*PDC1*::*LpDLDH* strain displaying the lowest Log_2_FC, indicating a more pronounced reduction in *FLO5* expression. This downregulation was consistent with the volcano plot analysis (Figure 9b), where *FLO5* was identified as one of the most significantly downregulated genes in response to the experimental conditions. Additionally, three genes (*LpDLDH*, *YGP1*, *SCW11*, and *SED1*) were upregulated. In contrast, the others were downregulated, indicating that integrating *LpDLDH* at the *CYB2* locus can facilitate a high expression of the *D-LDH* gene for D-LA production and enhance the expression of the *YGP1*, *SCW11*, and *SED1* genes involved in cell wall hydrophobicity (integrity), which is essential for maintaining cell robustness during fermentation.

These findings highlight the transcriptional impact of integrating the *D-LDH* gene into different loci, which may influence the efficiency of D-LA production and the metabolic balance in the engineered F118 strains.

## 4. Discussion

Previous studies have similarly reported that genomic loci can influence both transcriptional activity and metabolic pathway stability, further validating the strategic selection of these sites for metabolic engineering [29,30].

Among the engineered strains, only *LpDLDH* integrated into *CYB2*, *PDC1*, *PDC6*, and *MPC1* loci successfully produced D-LA, while other strains either failed to produce the desired metabolite or exhibited growth defects. Notably, strains with integrations into loci such as *DLD1*, *ADH1*, and *PDC5* loci either showed no D-LA production or did not grow in a medium containing antibiotics, possibly due to metabolic stress or the disruption of essential pathways. The differential production of D-LA, ethanol, and glycerol across strains highlights the influence of locus-specific metabolic fluxes. For example, strains that produced higher levels of D-LA often exhibited reduced ethanol production, suggesting a successful redirection of carbon flux away from the ethanol pathway. These findings are consistent with prior studies on ethanol pathway disruption, where metabolic rewiring resulted in the improved production of target metabolites [10,31,32]

The fermentation profiles of strains with gene disruptions but lacking the *LpDLDH* gene expression system revealed significant variations in growth and metabolite production. Strains disrupted in *PDC6* exhibited particularly slow growth and failed to complete fermentation, highlighting the essential role of this gene in glycolytic flux and redox balance. In contrast, the disruption of the *ADH1* gene resulted in reduced ethanol production, consistent with its central role in ethanol biosynthesis. Additionally, the non-producing strains exhibited a loss of flocculation, indicating a potential link between metabolic stress and cell wall integrity. Conversely, the high-producing Δ*PDC1*::*LpDLDH* strain exhibited reduced flocculation, which, while advantageous for fermentation homogeneity, may pose challenges for downstream processing. The producing strain, the Δ*PDC1*::*LpDLDH* strain (51 g/L of D-LA), exhibited reduced flocculation, and it has been reported that the replacement of *PDC1* with *LDH* to produce L-lactic acid was efficient [33].

The downregulation of the *FLO5* gene observed in most engineered D-LA-producing *S. cerevisiae* strains suggests a significant shift in cell wall integrity and flocculation behavior. The *FLO5* gene is a key gene within the *FLO* gene family, which encodes cell wall glycoproteins responsible for flocculation by mediating cell-cell adhesion via lectin-like interactions with mannose residues on adjacent cells [15]. The repression of *FLO5* gene expression in the engineered strains likely contributes to the loss of flocculant properties, leading to a more dispersed phenotype. A study about *Pichia pastoris* producing D-LA incorporated flocculation and showed the downregulation of cell wall rigidity [34].

Several studies have highlighted the role of the *FLO5* gene in determining the adhesive and flocculant characteristics of yeast cells. The study by [35] demonstrated that the overexpression of the *FLO5* gene enhances flocculation, whereas its deletion or downregulation reduces cell aggregation and increases sedimentation times. Other studies stated [36] that the specific adhesion properties are primarily determined by the characteristics of particular flocculin protein machinery rather than general cell wall properties, such as hydrophobicity. Each *FLO* gene expression contributes to distinct phenotypes and varying phenotype intensities, with *FLO1* and *FLO5* gene expressions responsible for cell aggregation and flocculation.

Additionally, environmental factors such as pH shifts and osmotic stress—conditions that arise during high D-LA production—are known to modulate *FLO5* gene expression [37]. The accumulation of D-LA in the fermentation medium likely contributes to stress responses, further driving the downregulation of flocculation genes as part of the yeast’s adaptation mechanism.

From an industrial perspective, the observed downregulation of *FLO5* gene expression may present advantages and challenges. Reduced flocculation can enhance bioprocess efficiency by promoting homogeneous mixing and improved substrate accessibility. However, it may also complicate downstream processing by increasing separation costs and reducing biomass retention in continuous fermentation systems. Future optimization strategies could involve fine-tuning *FLO5* gene expression using promoter engineering or laboratory adaptive evolution to balance production and flocculation properties.

The successful production of D-LA from glucose using engineered *S. cerevisiae* demonstrates the feasibility of utilizing genomic locus selection and transcriptomic insights for pathway optimization. Optimizing fermentation conditions, such as pH control and oxygen availability, could enhance yields and minimize byproducts like ethanol. Research by [38] employed an acid-tolerant strain to efficiently produce L-LA. A combination of metabolic engineering and fermentation regulation in these acid-tolerant strains proves beneficial for advancing LA production by *S. cerevisiae*. Additional challenges must be addressed before *S. cerevisiae* can become a commercially viable platform for renewable lactic acid production. Future engineering efforts aimed at increasing *S. cerevisiae*’s tolerance to low pH conditions and inhibitors present in common feedstock hydrolysates will be crucial for successful commercial applications [39]. The research conducted by [40] examined the transcriptional response of *S. cerevisiae* to LA enantiomers. The impact of pH on the D-LA response has highlighted the necessity of controlling and optimizing D-LA production in yeast under both neutralizing and non-neutralizing conditions separately.

Finally, further investigations into the relationship between metabolic stress, flocculation, and cell wall integrity under varying environmental conditions could provide a more comprehensive understanding of the cellular dynamics in engineered strains.

## 5. Conclusions

This study highlights the essential points for genetically engineering *S. cerevisiae* yeast strains to produce D-LA. Firstly, the integration locations of genes in the yeast genome are discussed. Only specific locations (*CYB2*, *PDC1*, *PDC6*, and *MPC1*) led to successful D-LA production. Secondly, the metabolic changes resulting from genetic modifications influenced how yeast cells generated various substances. Yeast strains that produced more D-LA often produced less ethanol, indicating a shift in related metabolism. Thirdly, the growth and fermentation processes of disrupted strains are analyzed. Some modified strains grew slowly or could not complete fermentation, emphasizing the significance of certain genes for yeast survival and function. Fourthly, changes in flocculation behavior are observed. Many D-LA-producing strains lost their ability to aggregate (flocculate), which was linked to the reduced expression of the *FLO5* gene, essential for cell–cell adhesion. Fifthly, industrial implications must be considered. The loss of flocculation may improve fermentation efficiency but could complicate separating yeast from the liquid afterward. Sixthly, as future directions, this study proposes methods to enhance D-LA production, such as fine-tuning gene expression, optimizing fermentation conditions, and further exploring the relationship between metabolic stress and cell behavior. Overall, this study illustrates how the careful selection of genetic modification sites and a thorough understanding of gene expression changes can help optimize yeast strains for producing desired substances like D-LA.

## Figures and Tables

**Table 1:** Summary of *D-LDH* transgene expression system’s integrated and disrupted strains.

Strain	Time ^a^ (hour)	Final D-LA (g/L)	Final Ethanol (g/L)	Final Glycerol (g/L)	Flocculation/ Hydrophobicity (%)	Final Dry Cell Weight (g/L)
Wild type	24	0.04 ± 0.03	40.63 ± 0.64	2.94 ± 0.26	Flocculant/89.32	6.31 ± 0.73
Δ*CYB2*::*LpDLDH*	12	31.92 ± 0.98 ***	31.64 ± 0.68 **	0	Flocculant/97.16	4.77 ± 0.22
Δ*PDC1*::*LpDLDH*	48	51.36 ± 1.78 ***	20.2 ± 1.69 **	0	Less flocculant/98.38	2.97 ± 0.12
Δ*MPC1*::*LpDLDH*	24	10.32 ± 0.35 ***	41.53 ± 0.22 ***	0	Flocculant/N.D.	6.96 ± 0.15
Δ*PDC6*::*LpDLDH*	24	0.026 ± 0.01 ***	45.11 ± 0.35 ***	4.25 ± 0.42 **	Flocculant/N.D.	9.42 ± 0.44
Δ*DLD1*::*LpDLDH*	48	0 *	33.84 ± 0.42 *	1.39 ± 0.05 ***	Non-flocculant/1.57	5.05 ± 0.059
Δ*ADH1*::*LpDLDH*	48	0 *	38.29 ± 2.66 *	1.31 ± 0.17 **	Non-flocculant/N.D.	6.9 ± 0.8
Δ*PDC5*::*LpDLDH*	48	0 *	41.69 ± 1.33 ***	1.39 ± 0.11 **	Non-flocculant/N.D.	6.63 ± 0.32
∆*CYB2*	24	0	45.96 ± 1.26	4.7 ± 1.15	Flocculant/N.D.	9.57 ± 1.5
∆*PDC1*	72	0	43.06 ± 1.07	4.41 ± 0.51	Flocculant/N.D.	3.95 ± 0.95
∆*MPC1*	21	0	45.72 ± 0.81	5.19 ± 0.61	Flocculant/N.D.	8.98 ± 0.17
∆*PDC6*	48	0	1.45 ± 2.43	0.57 ± 0.01	Less flocculant/N.D.	N.D. ^b^
∆*DLD1*	24	0	46.03 ± 1.19	3.95 ± 0.09	Flocculant/N.D.	9.26 ± 0.48
∆*ADH1*	144	0	39.07 ± 2.1 *	4.78 ± 0.21	Less flocculant/N.D.	3.71 ± 0.33
∆*PDC5*	48	0	44.61 ± 1.27	3.98 ± 0.11	Flocculant/N.D.	3.46 ± 0.37

^a^ fermentation time; ^b^ Δ*PDC6* strain exhibited incomplete fermentation, with slow growth and residual glucose (~100 g/L) remaining after 48 h. Statistical significance was assessed using Student’s *t*-test. Each value is the mean ± standard deviations (SD) (*n* = 3), * *p* < 0.05, ** *p* < 0.01, and *** *p* < 0.001. N.D., not determined.

## Data Availability

The original contributions presented in the study are included in the article/supplementary material; further inquiries can be directed to the corresponding authors.

## Supplementary Materials

The following supporting information can be downloaded at: https://www.mdpi.com/article/10.3390/microorganisms13030618/s1, Figure S1: Morphological changes in the shape of the WT, the Δ*CYB2*, and the Δ*CYB2*::*LpDLDH* strains after 48 h of cultivation in the YPD100 medium without the addition of neutralizer; Figure S2: Morphological changes on the shape of the F118 strain cells with locus destruction only (a) and concomitantly with the integration of the *LpDLDH* transgene expression cassette (b) after 48 h of cultivation in the YPD100 medium without the addition of neutralizer, confirmed by using bright light mode; Table S1: Primers used for the construction of D-LA engineered strains; Table S2: Primers used for the construction of disrupted strains; Table S3: Primers used in qPCR analysis; Table S4: Ct value of housekeeping genes expression in engineered F118 strains.

## Abbreviations

The following abbreviations are used in this manuscript:

LA	Lactic acid
*D-LDH*	D-lactate dehydrogenase
*CYB2*	Cytochrome b2
*PDC1/5/6*	Pyruvate decarboxylase
*DLD1*	D-lactate dehydrogenase
*MPC1*	Mitochondrial pyruvate carrier
*ADH1*	Alcohol dehydrogenase
*GAP*	Glyceraldehyde 3-phosphate
*DHAP*	Dihydroxyacetone phosphate
*G3P*	Glycerol 3-phosphate
Ct	Threshold cycle
Log_2_FC	Log fold change

## References

[1] Tan J., Abdel-Rahman M.A., Sonomoto K., Di Lorenzo M.L., Androsch R. (2017). Biorefinery-Based Lactic Acid Fermentation: Microbial Production of Pure Monomer Product. Synthesis, Structure and Properties of Poly (Lactic Acid).

[2] Gallezot P. (2012). Conversion of Biomass to Selected Chemical Products. Chem. Soc. Rev..

[3] Abdel-Rahman M.A., Tashiro Y., Sonomoto K. (2011). Lactic Acid Production from Lignocellulose-Derived Sugars Using Lactic Acid Bacteria: Overview and Limits. J. Biotechnol..

[4] Porro D., Brambilla L., Ranzi B.M., Martegani E., Alberghina L. (1995). Development of Metabolically Engineered *Saccharomyces Cerevisiae* Cells for the Production of Lactic Acid. Biotechnol. Prog..

[5] Park H.J., Bae J., Ko H., Lee S., Sung B.H., Han J., Sohn J. (2018). Low-pH Production of D-lactic Acid Using Newly Isolated Acid Tolerant Yeast *Pichia kudriavzevii* NG7. Biotechnol. Bioeng..

[6] Abdel-Rahman M.A., Sonomoto K. (2016). Opportunities to Overcome the Current Limitations and Challenges for Efficient Microbial Production of Optically Pure Lactic Acid. J. Biotechnol..

[7] Lahtinen S., Ouwehand A.C., Salminen S., Von Wright A. (2011). Lactic Acid Bacteria: Microbiological and Functional Aspects.

[8] Bai H., Deng S., Bai D., Zhang Q., Fu Q. (2017). Recent Advances in Processing of Stereocomplex-Type Polylactide. Macromol. Rapid Commun..

[9] Coelho L.F., De Lima C.J.B., Bernardo M.P., Contiero J. (2011). D(−)-Lactic Acid Production by *Leuconostoc mesenteroides* B512 Using Different Carbon and Nitrogen Sources. Appl. Biochem. Biotechnol..

[10] Baek S.-H., Kwon E.Y., Kim Y.H., Hahn J.-S. (2016). Metabolic Engineering and Adaptive Evolution for Efficient Production of D-Lactic Acid in *Saccharomyces cerevisiae*. Appl. Microbiol. Biotechnol..

[11] Sauer M., Porro D., Mattanovich D., Branduardi P. (2010). 16 Years Research on Lactic Acid Production with Yeast–Ready for the Market?. Biotechnol. Genet. Eng. Rev..

[12] He L.-Y., Zhao X.-Q., Bai F.-W. (2012). Engineering Industrial Saccharomyces Cerevisiae Strain with the FLO1-Derivative Gene Isolated from the Flocculating Yeast SPSC01 for Constitutive Flocculation and Fuel Ethanol Production. Appl. Energy.

[13] Kahar P., Itomi A., Tsuboi H., Ishizaki M., Yasuda M., Kihira C., Otsuka H., Azmi N.B., Matsumoto H., Ogino C. (2022). The Flocculant *Saccharomyces cerevisiae* Strain Gains Robustness via Alteration of the Cell Wall Hydrophobicity. Metab. Eng..

[14] Daniel Gietz R., Woods R.A. (2002). Transformation of Yeast by Lithium Acetate/Single-Stranded Carrier DNA/Polyethylene Glycol Method. Methods in Enzymology.

[15] Van Mulders S.E., Christianen E., Saerens S.M.G., Daenen L., Verbelen P.J., Willaert R., Verstrepen K.J., Delvaux F.R. (2009). Phenotypic Diversity of Flo Protein Family-Mediated Adhesion in *Saccharomyces cerevisiae*. FEMS Yeast Res..

[16] Ramil E., Agrimonti C., Shechter E., Gervais M., Guiard B. (2000). Regulation of the *CYB2* Gene Expression: Transcriptional Co-ordination by the Hap1p, Hap2/3/4/5p and Adr1p Transcription Factors. Mol. Microbiol..

[17] Ookubo A., Hirasawa T., Yoshikawa K., Nagahisa K., Furusawa C., Shimizu H. (2008). Improvement of L-Lactate Production by *CYB2* Gene Disruption in a Recombinant *Saccharomyces cerevisiae* Strain under Low pH Condition. Biosci. Biotechnol. Biochem..

[18] Pangestu R., Kahar P., Ogino C., Kondo A. (2024). Comparative Responses of Flocculating and Nonflocculating Yeasts to Cell Density and Chemical Stress in Lactic Acid Fermentation. Yeast.

[19] Carmelo V., Santos H., Sá-Correia I. (1997). Effect of Extracellular Acidification on the Activity of Plasma Membrane ATPase and on the Cytosolic and Vacuolar pH of *Saccharomyces cerevisiae*. Biochim. Biophys. Acta BBA-Biomembr..

[20] Ambesi A., Miranda M., Petrov V.V., Slayman C.W. (2000). Biogenesis and Function of the Yeast Plasma-Membrane H+-ATPase. J. Exp. Biol..

[21] Morsomme P., Slayman C.W., Goffeau A. (2000). Mutagenic Study of the Structure, Function and Biogenesis of the Yeast Plasma Membrane H+-ATPase. Biochim. Biophys. Acta BBA-Rev. Biomembr..

[22] Fernandes A.R., Mira N.P., Vargas R.C., Canelhas I., Sá-Correia I. (2005). Saccharomyces Cerevisiae Adaptation to Weak Acids Involves the Transcription Factor Haa1p and Haa1p-Regulated Genes. Biochem. Biophys. Res. Commun..

[23] Mira N.P., Becker J.D., Sá-Correia I. (2010). Genomic Expression Program Involving the Haa1p-Regulon in *Saccharomyces cerevisiae* Response to Acetic Acid. OMICS J. Integr. Biol..

[24] Branduardi P., Sauer M., De Gioia L., Zampella G., Valli M., Mattanovich D., Porro D. (2006). Lactate production yield from engineered yeasts is dependent from the host background, the lactate dehydrogenase source and the lactate export. Microb. Cell Factories.

[25] Zhong W., Yang M., Mu T., Wu F., Hao X., Chen R., Sharshar M.M., Thygesen A., Wang Q., Xing J. (2019). Systematically Redesigning and Optimizing the Expression of D-Lactate Dehydrogenase Efficiently Produces High-Optical-Purity D-Lactic Acid in *Saccharomyces cerevisiae*. Biochem. Eng. J..

[26] Tokuhiro K., Ishida N., Nagamori E., Saitoh S., Onishi T., Kondo A., Takahashi H. (2009). Double Mutation of the PDC1 and ADH1 Genes Improves Lactate Production in the Yeast *Saccharomyces cerevisiae* Expressing the Bovine Lactate Dehydrogenase Gene. Appl. Microbiol. Biotechnol..

[27] Van Maris A.J.A., Geertman J.-M.A., Vermeulen A., Groothuizen M.K., Winkler A.A., Piper M.D.W., Van Dijken J.P., Pronk J.T. (2004). Directed Evolution of Pyruvate Decarboxylase-Negative *Saccharomyces cerevisiae*, Yielding a C_2_-Independent, Glucose-Tolerant, and Pyruvate-Hyperproducing Yeast. Appl. Environ. Microbiol..

[28] Bender T., Pena G., Martinou J. (2015). Regulation of Mitochondrial Pyruvate Uptake by Alternative Pyruvate Carrier Complexes. EMBO J..

[29] Kong S., Yu W., Gao N., Zhai X., Zhou Y.J. (2022). Expanding the Neutral Sites for Integrated Gene Expression in *Saccharomyces cerevisiae*. FEMS Microbiol. Lett..

[30] Wu X.-L., Li B.-Z., Zhang W.-Z., Song K., Qi H., Dai J., Yuan Y.-J. (2017). Genome-Wide Landscape of Position Effects on Heterogeneous Gene Expression in *Saccharomyces cerevisiae*. Biotechnol. Biofuels.

[31] Watcharawipas A., Sae-tang K., Sansatchanon K., Sudying P., Boonchoo K., Tanapongpipat S., Kocharin K., Runguphan W. (2021). Systematic Engineering of *Saccharomyces cerevisiae* for D-Lactic Acid Production with near Theoretical Yield. FEMS Yeast Res..

[32] Zhu P., Luo R., Li Y., Chen X. (2022). Metabolic Engineering and Adaptive Evolution for Efficient Production of l-Lactic Acid in *Saccharomyces cerevisiae*. Microbiol. Spectr..

[33] Ishida N., Saitoh S., Ohnishi T., Tokuhiro K., Nagamori E., Kitamoto K., Takahashi H. (2006). Metabolic Engineering of *Saccharomyces cerevisiae* for Efficient Production of Pure L-(+)-Lactic Acid. Appl. Biochem. Biotechnol..

[34] Sae-Tang K., Bumrungtham P., Mhuantong W., Champreda V., Tanapongpipat S., Zhao X.-Q., Liu C.-G., Runguphan W. (2023). Engineering Flocculation for Improved Tolerance and Production of D-Lactic Acid in Pichia Pastoris. J. Fungi.

[35] Goossens K.V.Y., Ielasi F.S., Nookaew I., Stals I., Alonso-Sarduy L., Daenen L., Van Mulders S.E., Stassen C., Van Eijsden R.G.E., Siewers V. (2015). Molecular Mechanism of Flocculation Self-Recognition in Yeast and Its Role in Mating and Survival. mBio.

[36] Govender P., Domingo J.L., Bester M.C., Pretorius I.S., Bauer F.F. (2008). Controlled Expression of the Dominant Flocculation Genes *FLO1*, *FLO5*, and *FLO11* in *Saccharomyces cerevisiae*. Appl. Environ. Microbiol..

[37] Soares E.V. (2011). Flocculation in *Saccharomyces cerevisiae*: A Review: Yeast Flocculation: A Review. J. Appl. Microbiol..

[38] Liu T., Sun L., Zhang C., Liu Y., Li J., Du G., Lv X., Liu L. (2023). Combinatorial Metabolic Engineering and Process Optimization Enables Highly Efficient Production of L-Lactic Acid by Acid-Tolerant *Saccharomyces cerevisiae*. Bioresour. Technol..

[39] Lane S., Turner T.L., Jin Y. (2023). Glucose Assimilation Rate Determines the Partition of Flux at Pyruvate between Lactic Acid and Ethanol in *Saccharomyces cerevisiae*. Biotechnol. J..

[40] Drozdova P., Gurkov A., Saranchina A., Vlasevskaya A., Zolotovskaya E., Indosova E., Timofeyev M., Borvinskaya E. (2024). Transcriptional Response of *Saccharomyces cerevisiae* to Lactic Acid Enantiomers. Appl. Microbiol. Biotechnol..

