# Supplementary material for: Factors Affecting D-Lactic Acid Production by Flocculant Saccharomyces cerevisiae Under Non-Neutralizing Conditions
Source: Microorganisms. 2025 Mar 7;13(3):618. doi: 10.3390/microorganisms13030618 (PMC11944911; doi:10.3390/microorganisms13030618)
Supplement: Supplementary file 1 [file microorganisms-13-00618-s001.zip › microorganisms-3484817-supplementary.pdf]

Supplementary Information

Factors Affecting D-Lactic Acid Production by Flocculant *Saccharomyces cerevisiae* Under Non-Neutralizing Conditions

Dianti Rahmasari [1,†], Prihardi Kahar [1,2,3,*,†], Arthur Vinícius de Oliveira [1], Filemon Jalu Nusantara Putra [1], Akihiko Kondo [3] and Chiaki Ogino [1,2,4,*]

[1] Department of Chemical Science and Engineering, Graduate School of Engineering, Kobe University,
Kobe 658-8501, Hyogo, Japan; dianti.rahmasari@gmail.com (D.R.); arthurolv20@gmail.com (A.V.D.O.); jalu@bear.kobe-u.ac.jp (F.J.N.P.)

[2] Engineering Biology Research Center, Kobe University, 1-1 Rokkodai-cho, Nada-Ku, Kobe 657-8501, Hyogo, Japan

[3] Graduate School of Science, Technology, and Innovation (STIN), Kobe University, Kobe 658-8501, Hyogo, Japan; akondo@kobe-u.ac.jp

[4] Research Center for Membrane and Film Technology, Kobe University, Kobe 657-8501, Hyogo, Japan

* Correspondence: pri@port.kobe-u.ac.jp (P.K.); ochiaki@port.kobe-u.ac.jp (C.O.)

† These authors contributed equally to this work.

Figure S1. Morphological changes in the shape of the WT, the Δ*CYB2*, and the Δ*CYB2::LpDLDH* strains after 48 hours of cultivation in the YPD100 medium without the addition of neutralizer.

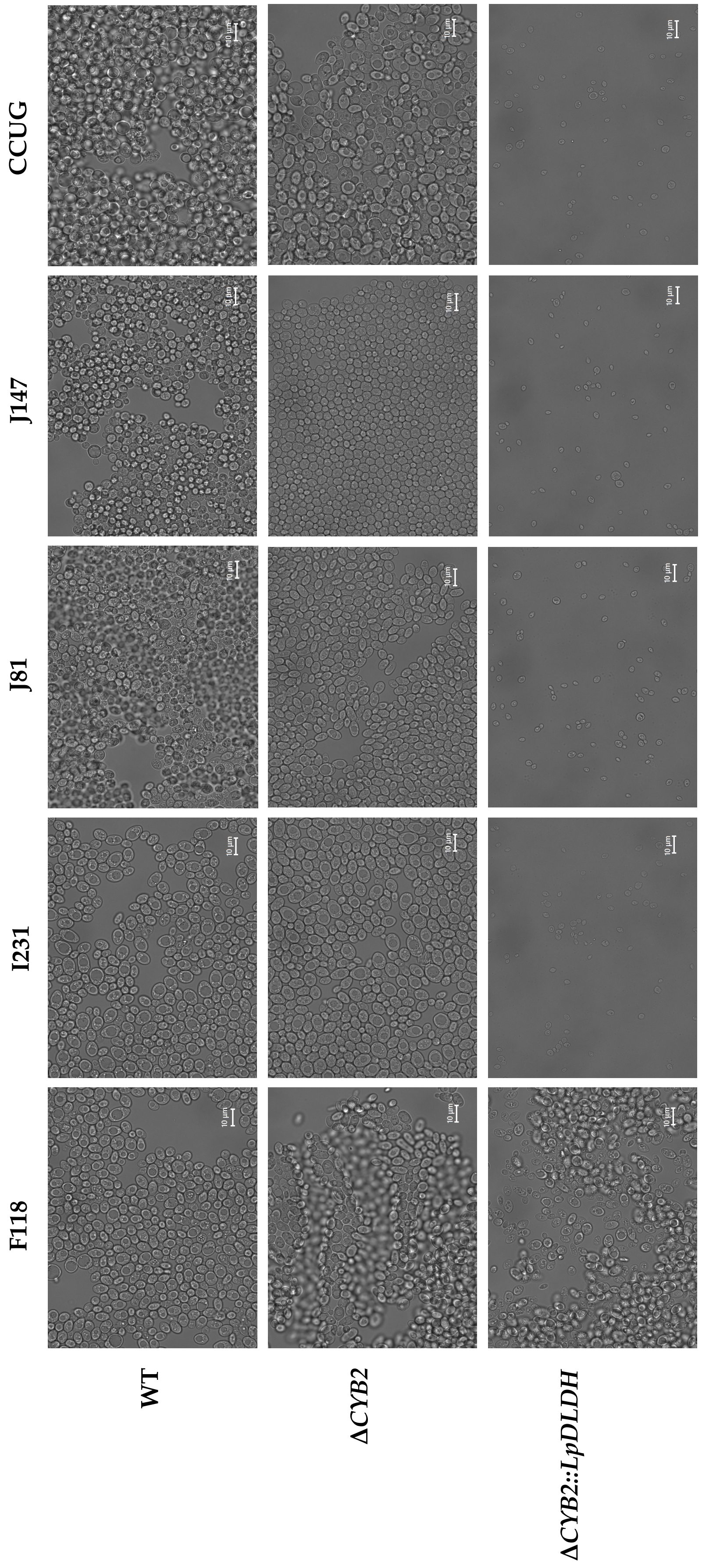

Figure S2. Morphological changes on the shape of the F118 strain cells with locus destruction only (a) and concomitantly with the integration of *LpDLDH* transgene expression cassette (b) after 48 h of cultivation in the YPD100 medium without the addition of neutralizer, confirmed by using bright light mode.

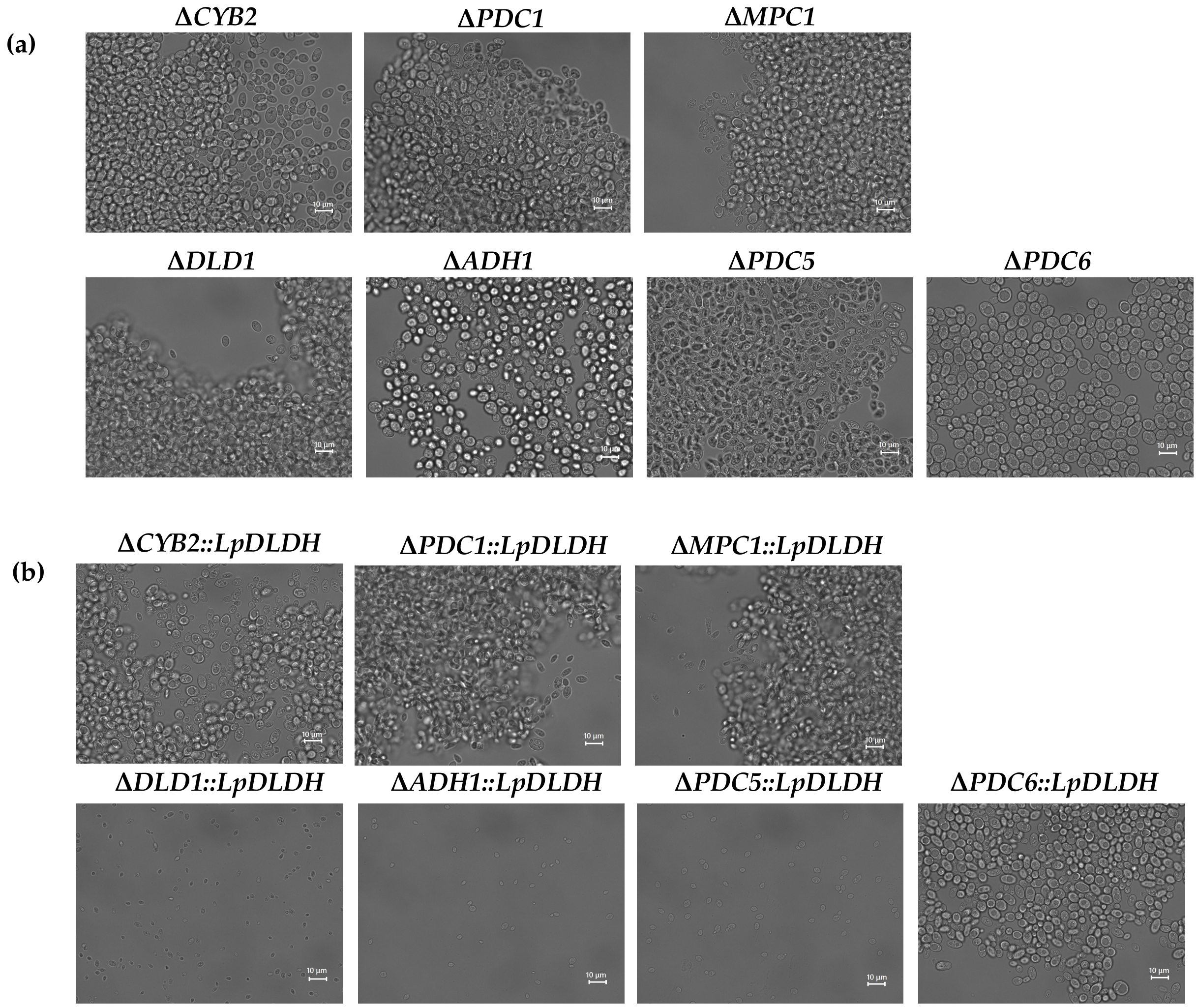

Table S1. Primers used for the construction of D-LA engineered strains.

Strain	Primer	Sequence 5′ → 3′	Description
All strains	LpDLDH.FOR	5'CGAATAAACACACATAAATAAACAAAATGAAGATTTTCGCCTACGGTATCAGAGATGACG3′	Amplifying *LpDLDH* gene from codon optimized *Leuconostoc pseumesenteroides D-LDH*
	LpDLDH.REV	5'AAGTAAATTCACTTAGTACTTAACGGCAATAGCTGGAGTTTCACCTTTAGCAAAAG3′	
Δ*CYB2*::*LpDLDH*	CYB2Left.FOR	5'ATGCTAAAATACAAACCTTTACTAAAAATCTCGAAGAACTGTGAGGCTGCTATCCTCAGAGCG3′	Amplifying partial region of *CYB2* gene (left part) from F118 genome as template
	CYB2Left.REV	5'ATACGAAGTTATAGGTAGCAGCGATTTTAGTTGTTCTTT3′	
	CYB2Right.FOR	5'TATTGATAATGAAGCGTTATCAAAGTTTATTGACCCCT3′	Amplifying partial region of *CYB2* gene (right part) from F118 genome as template
	CYB2Right.REV	5'TCATGCATCCTCAAATTCTGTTAAAGTAGGTCCCTCATAAACTTCATTATACAGCACGTCG3′	
	CYB2-KanMX-DLDH-CYB2.FOR	5'GTAAAGTTCTTCTTCCAGATTGTGCGCAAACTCTTGCGATATGCTAAAATACAAACCTTTACTAAAAATCTCGAAGAACTGTGAGGCTGC3′	Amplifying region *CYB2Left-Kmx-DLDH-CYB2Right* overhang with plasmid pAUR101
	CYB2-KanMX-DLDH-CYB2.REV	5'AGGGGTTCCGCGCACATTTCCCCGAAAAGTGCCACCTGACTCATGCATCCTCAAATTCTGTTAAAGTAGGTCCCTCATAAACT3′	
	loxpC-KanMX-loxpC.FOR	5'TCGCTGCTACCTATAACTTCGTATAGCATACATTATACGAAGTTATATTAAGGG3′	Amplifying region *loxp-KanMX-loxP* overhang with partial region of *CYB2* left and right
	loxpC-KanMX-loxC.REV	5'CATATCTTTCCCTATAACTTCGTATAATGTATGCTATACGAAGTTATTAGGTCT3′	
Δ*PDC1*::*LpDLDH*	PDC1Left.FOR	5'ATGTCTGAAATTACTTTGGGTAAATATTTGTTCGAAAGATTAAAGCAAGTCAACGTTAACACCGTTTTCG3′	Amplifying partial region of *PDC1* gene (left part) from F118 genome as template
	PDC1Left.REV	5'GACTTCCTTTTCGGATTCAGCATCGTTTGGCTTCAAAGACATGTC3′	
	PDC1Right.FOR	5'GTGGAACCAATTGGGTAACTTCTTGCAAGAAGGTGATGTTGTC 3′	Amplifying partial region of *PDC1* gene (right part)

	PDC1Right.REV	5'TTATTGCTTAGCGTTGGTAGCAGCAGTCAACTTAGCTTGTTCA3′	from F118 genome as template
	PDC1-KanMX-DLDH-PDC1.FOR	5'GTAAAGTTCTTCTTCCAGATTGTGCGCAAACTCTTGCGATATGTCTGAAATTACTTTGGGTAAATATTTGTTCGAAAGATTAAAGCAAG3′	Amplifying region *PDC1Left-Kmx-DLDH-PDC5Right* overhang with plasmid
	PDC1-KanMX-DLDH-PDC1.REV	5'CGAAAAGTGCCACCTGACTTATTGCTTAGCGTTGGTAGCAGCAGTCAACTTAGCTTGTTCAACCAAG3′	
	loxpP-KanMX-loxP.FOR	5'CTGAATCCGAAAAGGAAGTCATAACTTCGTATAGCATACATTATACGAAGTTATAT3′	Amplifying region *loxp-KanMX-loxP* overhang with partial region of *PDC1* left and right
	loxpP-KanMX-loxP.REV	5'CGAAAAGTGCCACCTGACTTATTGCTTAGCGTTGGTAGCAGCAGTCAACTTAGCTTGTTCAACCAAG3′	
Δ*DLD1*::*Lp DLDH*	DLD1.FOR	5′ AACAGGCACAGGCAGCACCGACAGCAG3′	Amplifying partial region of *DLD1* gene from F118 genome as template
	DLD1.REV	5′ TGGTATCTCCAATTCTTGTAGGCAGGAAGTGCCCCTCCA3′	
	DLD1-KanMX-DLDH-ADH1.REV	5′ GGCCGCATGGCCGCGTACGCTGCAGGTCGACGGATCTGATAACAGGCACAGGCAGCACCGACAGCAG3′	Amplifying region *DLD1Left-Kmx-DLDH-DLD1Right* overhang with plasmid
	DLD1-KanMX-DLDH-ADH1.REV	5′ AACTTCGTATAATGTATGCTATACGAAGTTATTAGGTGATTGGTATCTCCAATTCTTGTAGGCAGGAAGTGCCCCTCCA3′	
Δ*ADH1*::*Lp DLDH*	ADH1Left.FOR	5'GCTTGGCACGGTGACTGGCCATTGCCAGTTAAGC3′	Amplifying partial region of *ADH1* gene (left part) from F118 genome as template
	ADh1Left.REV	5'CTGTTCAAGCCGCTCACATTATAACTTCGTATAGCATACATTATACGAAGTTATATTAAGGGTTCTCGAC3′	
	ADH1Right.FOR	5'GGCCCAAGTCGCCCCCATCTTGTGTGC3′	Amplifying partial region of *ADH1* gene (right part) from F118 genome as template
	ADH1Right.REV	5'ACCACCGATGGATCTGAATAATTCTTCCTTACCTTCAC3′	

	ADH1-KanMX-DLDH-ADH1.FOR	5'ATTTCCCCGAAAAGTGCCACCTGACACCACCGATGGATCTGAATAATTCTTCCTTACCTTCA3′	Amplifying region *ADH1Left-Kmx-DLDH-ADH1Right* overhang with plasmid
	ADH1-KanMX-DLDH-ADH1.REV	5'ATTTCCCCGAAAAGTGCCACCTGACACCACCGATGGATCTGAATAATTCTTCCTTACCTTCA3′	
	loxpA-KanMX-loxpA.FOR	5'CTGTTCAAGCCGCTCACATTATAACTTCGTATAGCATACATTATACGAAGTTATATTAAGGGTTCTCGAC3′	Amplifying region *loxp-KanMX-loxP* overhang with partial region of *ADH1* left and right
	loxpA-KanMX-loxpA.REV	5′TGTATGCTATACGAAGTTATTAACTTGGCTGGGACGTTCAAGTCAACCAAGTTAGCTGGC3′	
Δ*PDC5*::*Lp DLDH*	PDC5Left.FOR	5'GTGAATTGTCTGCTTTGAATGGTATTGCCGGTTCTTACG3′	Amplifying partial region of *PDC5* gene (left part) from F118 genome as template
	PDC5Left.REV	5'TGTATGCTATACGAAGTTATTAACTTGGCTGGGACGTTCAAGTCAACCAAGTTAGCTGGC3′	
	PDC5Right.FOR	5'GTTATTGGAAACTCCAATTGACTTGTCTTTGAAGCCAAACGACG3′	Amplifying partial region of *PDC5* gene (right part) from F118 genome as template
	PDC5Right.REV	5'ATGTGGTCAGAGTGGAATTCAACGATATTTTTGGTCTTGTAG3′	
	PDC5-KanMX-DLDH-PDC5.FOR	5'AGATTGTGCGCAAACTCTTGCGATGTGAATTGTCTGCTTTGAATGGTATTGCC3′	Amplifying region *PDC5Left-Kmx-DLDH-PDC5Right* overhang with plasmid
	PDC5-KanMX-DLDH-PDC5.REV	5'ATTTCCCCGAAAAGTGCCACCTGACATGTGGTCAGAGTGGAATTCAACGATATTTTTGGTC3′	
	loxpP5-KanMXloxpP5.FOR	5'TGAACGTCCCAGCCAAGTTAATAACTTCGTATAGCATACATTATACGAAGTTATATTAAGGGTTCTCGAC3′	Amplifying region *loxp-KanMX-loxP* overhang with partial region of PDC5 left and right

	loxpP5-KanMXloxpP5.REV	5'TGTATGCTATACGAAGTTATTAACTTGGCTGGGACGTTCAAGTCAACCAAGTTAGCTGGC3′	
ΔMPC1::LpDLDH	MPC1.FOR	5'GGCCGCATGGCCGCGTACGCTGCAGGTCGACGGATCTGATATGTCTCAACCGGTTCAACGCGCTGCAGC3′	Amplifyng partial region of *MPC1* gene from F118 genome as template overhang with plasmid and *Kmx- DLDH* region
	MPC1.REV	5'AACTTCGTATAATGTATGCTATACGAAGTTATTAGGTGATTTACTGTTTACCAGTTTTTTCTTTCTCTTTCCATTCCTTATCTAGAGCTTTCTTCTCC3′	
	GAP-DLDH-T.FOR	5'GACGACGACGATAAGGATTACAAGGACGACGACGATAAAGAGGGAAAGATATGAGCTATACAGCGGAATTTCTATATCACTCAGATTTTG3′	Amplifying *Promoter-DLDH-Terminator* region overhang with *MPC1-Kmx* and plasmid
	GAP-DLDH-T.REV	5'AGTGAAAGGAAGGCCCATGAGGCCCAGCTCTGTCGCCATGTCATTATCAATACTGCCATTTCAAAGAATACGTAAATAATTAATAGTAGTGATTTTCC3′	
ΔPDC6::LpDLDH	PDC6.FOR	5'GGCCGCATGGCCGCGTACGCTGCAGGTCGACGGATCTGATAAGGGTTTATCTGTGCTGGTAACTACTTTTGGCGTAGGTGAATTATCCG3′	Amplifyng partial region of *PDC6* gene from F118 genome as template overhang with plasmid and *Kmx-DLDH* region
	PDC6.REV	5'AACTTCGTATAATGTATGCTATACGAAGTTATTAGGTGATATTGACCCTTTACCTAGAGGTGTCACAAAAGCTGGGAATTGCGTCA3′	
	GAP-DLDH-T.FOR	5'GACGACGACGATAAGGATTACAAGGACGACGACGATAAAGAGGGAAAGATATGAGCTATACAGCGGAATTTCTATATCACTCAGATTTTG3′	Amplifying *Promoter-DLDH-Terminator* region overhang with *PDC6-Kmx* and plasmid
	GAP-DLDH-T.REV	5'AGTGAAAGGAAGGCCCATGAGGCCCAGCTCTGTCGCCATGTCATTATCAATACTGCCATTTCAAAGAATACGTAAATAATTAATAGTAG3′	

Table S2. Primers used for the construction of disrupted strains.

Strain	Primer name	Sequence (5' -->3′	Description
Δ*CYB2*	CYB2.FOR	5'TAGATTGAACACAATCCGCGCGTACGGTTCTACCGT3′	Amplifying partial region of *CYB2* gene from F118 genome as template
	CYB2.REV	5'TGTTCTTTTCTAGCGATATCTTCCTTAGTTTCACCAGGAGCATAAGGAGG3′	
	CYB2-Kmx.FOR	5'GGCCGCATGGCCGCGTACGCTGCAGGTCGACGGATCTGATTAGATTGAACACAATCCGCGCGTACGGTTCTACCGTTCC3′	Amplifying partial *CYB2-Kmx* overhang with plasmid and *loxp*
	CYB2-Kmx.REV	5'AACTTCGTATAATGTATGCTATACGAAGTTATTAGGTGATTGTTCTTTTCTAGCGATATCTTCCTTAGTTTCACCAGGAGCATAAGGAGG3′	
Δ*PDC1*	PDC1-Kmx.FOR	5'GGCCGCATGGCCGCGTACGCTGCAGGTCGACGGATCTGATATTAAAGCAAGTCAACGTTAACACCGTTTTCGGTTTGCCAGGTGAC3′	Amplifying partial *PDC1-Kmx* overhang with plasmid and *loxp*
	PDC1-Kmx.REV	5'AACTTCGTATAATGTATGCTATACGAAGTTATTAGGTGATAATGACTTCCTTTTCGGATTCAGCATCGTTTGGCTTCAAAGACATGT3′	
Δ*DLD1*	DLD1.FOR	5'AACAGGCACAGGCAGCACCGACAGCAG3′	Amplifying partial region of *DLD1* gene from F118 genome as template
	DLD1.REV	5'TGGTATCTCCAATTCTTGTAGGCAGGAAGTGCCCCTCCA3′	
	DLD1-Kmx.FOR	5'GGCCGCATGGCCGCGTACGCTGCAGGTCGACGGATCTGATAACAGGCACAGGCAGCACCGACAGCAG3′	Amplifying partial *DLD1-Kmx* overhang with plasmid and *loxp*
	DLD1-Kmx.REV	5'AACTTCGTATAATGTATGCTATACGAAGTTATTAGGTGATTGGTATCTCCAATTCTTGTAGGCAGGAAGTGCCCCTCCA3′	
Δ*ADH1*	ADH1-Kmx.FOR	5'GGCCGCATGGCCGCGTACGCTGCAGGTCGACGGATCTGATGCTTGGCACGGT3′	Amplifying partial *ADH1-Kmx* overhang with plasmid and loxp
	ADH1-Kmx.REV	5'AACTTCGTATAATGTATGCTATACGAAGTTATTAGGTGATACCACCGATGGATCTGAATAATTCTTCCTTACCTTCACCACCG3′	
Δ*PDC5*	PDC5-Kmx.FOR	5'GGCCGCATGGCCGCGTACGCTGCAGGTCGACGGATCTGATCAGCTAACTTGGTTGACTTGAACGTCCCAGCCAAGTTATTGGAAACTCC3′	Amplifying partial *PDC5-Kmx* overhang with plasmid and *loxp*

	PDC5-Kmx.REV	5'AACTTCGTATAATGTATGCTATACGAAGTTATTAGGTGATGTAGTCCTTGACGACTTCTGGAATAGCATCCAACAATTTTTGCAAGGCA3′	
Δ*PDC6*	PDC6-Kmx.FOR	5'GGCCGCATGGCCGCGTACGCTGCAGGTCGACGGATCTGATAAGGGTTTATCTGTGCTGGTAACTACTTTTGGCGTAGGTGAATTATCCG3′	Amplifying partial *PDC6-Kmx* overhang with plasmid and *loxp*
	PDC6-Kmx.REV	5'AACTTCGTATAATGTATGCTATACGAAGTTATTAGGTGATATTGACCCTTTACCTAGAGGTGTCACAAAAGCTGGGAATTGCGTCA3′	
Δ*MPC1*	MPC1-Kmx.FOR	5'GGCCGCATGGCCGCGTACGCTGCAGGTCGACGGATCTGATATGTCTCAACCG3′	Amplifying partial *MPC1-Kmx* overhang with plasmid pPC01 and *loxp*
	MPC1-Kmx.REV	5'AACTTCGTATAATGTATGCTATACGAAGTTATTAGGTGATTTACTGTTTACCAGTTTTTTCTTTCTCTTTCCATTCCTTATCTAGAGC3′	

Table S3. Primers used in qPCR analysis.

Gene	Forward primer (5' →3')	Reverse primer (5'→3')
LpDLDH	AATGTTCCAGTCTACTCCCC	CGATAACTTTAGCACCAAAACC
*TUB1**	ATACCACATCCATTGCTGAG	ATACCTTCACCGACATACCA
*ACT1**	GCCGAAAGAATGCAAAAGGA	TAGAACCACCAATCCAGACG
ADH1	GGCTATGGGTTACAGAGT	TTCCTTAGTGAAGTCAATGAAG
PDC1	ACACCATCTTGGCTTTGGTC	CGAAAGCTGGGAATTGAGTC
PDC5	CACGTTGTTGGTGTTCCATC	TCAGTGATCATGGCAGTGGT
PDC6	GGAGATTGACCCCAACAAGA	ATACGGCTTTAACCCCCATC
FLO5	TCTTCCTCTTCTATCTCTTCTGA	GGTAATGATGAAGTGATTTCCTG
HSP150	TCTGAGCCTTGGTCCACTTT	ATGATGCGCTGGATGTAGTG
DSE2	AACAGCAATGGGGAAACAG	GTAGTGGAAGAAACGACGG
SCW10	CTTCATCCTCACCCTCATCC	GCAAGACATTCTCAACCTGAC
SCW11	CAAGGTGTGAACCCAACAG	GGAAGGAGAGGAGGAGATAGAG
HOG1	TGCCGAAATGATTGAAGGTAAG	AGGAGCCGAATAAGGATGAG
YGP1	CTCCTTGAGTTCTGCCGTTC	TGGGGATAGGTAACCAGCAC
TOS6	TTACCACCACCCCACAAAG	ACAAAGCAAAGGCAGCAG
SED1	CCAACAACCACATCAACCAC	GAGAAGAAGCAGAGGATGAAAC
CRH1	GTATGACCAAGCCCAAGAAG	GAAGATGGAGATGTGGATGAAG
AFT1	TCACCAAAATCAGCCCCAC	CTTCACATACTTCAACTCC
YAF9	CGTTAAACCACCAAATGCTCC	ATTCACCCCACCCTGTTTC
SSK1	ACCAAACCTTTTTACCACCAC	CAGCTTCTATACCAGACAAGAC
CTS2	TCATTTCCCTCACGACATAAAC	TTCACTATCAGACCAGCCC
EAF5	AACTACGACTACGCCACAC	ATGACCTTTCGCATTACACC
EAF6	TGAAGCATTGAAAGCGGAAC	TGGTGTGACTTGGAGAAGG
CYT1	ATGGTTGAGTACGAAGATGG	TTCTTTCGTCATGTTCAGGT
SUN4	AAATCGCTACCACCTCGTC	CCAGCCACCTTCATCTAACC
EGT2	CTCCAACTCCACAACATCATC	GAATATAAGACCCAGAGGCAAC
TPS1	ACAACGAGGCAAACCAGAC	GGAACCCGACTAAATCACAAC
PBS2	CAAAGCACATCCACCTCATC	ACCACCACCACCACTACTAC
VMA2	CGCTGAATACCTTGCTTACC	GTTACGACCCTCTACTCTACC
VMA6	TGCAAAGTTCAGATATTGACCC	GTTACCAGTCTCCAAAAATCCC
NDI1	TTGGGACAGTAGACGAAAAG	GGATTGATAGAGGTGGCTTC
JEN1	GGCTACTTTAGGGATGATGG	CGTTTCAGGCCATAACAATC

*Housekeeping genes used in this study.

Table S4. Ct value of housekeeping genes expression in engineered F118 strains.

Strains	D-LA production	C_t value	
		**TUB1**	**ACT1**
Wildtype	No (control)	24.91	22.48
Δ*CYB2::LpDLDH*	Yes	25.51	22.7
Δ*PDC1::LpDLDH*	Yes	27.73	22.53
Δ*PDC6::LpDLDH*	Yes	N.D.	21.37
Δ*MPC1::LpDLDH*	Yes	N.D.	20.49
Δ*DLD1::LpDLDH*	No	N.D.	30.01
Δ*ADH1::LpDLDH*	No	N.D.	32.11
Δ*PDC5::LpDLDH*	No	N.D.	32.45

N.D., not determined.
